# Development of a mixed-effects longitudinal spline regression method for growth data analysis

**DOI:** 10.1016/j.mex.2026.104164

**Published:** 2026-09-13

**Authors:** Isra Leyla Bangsawang, Anna Islamiyati, Erna Tri Herdiani

**Affiliations:** Department of Statistics, Faculty of Mathematical and Natural Sciences, Hasanuddin University, Makassar, 90245, Indonesia

**Keywords:** Cubic spline, Growth curve, Longitudinal data, Mixed effects

## Abstract

Conventional parametric regression in longitudinal studies frequently violates the homoscedasticity and linearity assumptions. Moreover, in practice, it is common to observe data patterns that are not defined by any specific parametric functional form. As a result, the parametric longitudinal regression model risks a biased estimation. Therefore, this study proposes the development of a mixed-effects longitudinal model that incorporates a spline estimator. The proposed method is applied to analyze the growth of children’s weight based on age and height. This method simultaneously accommodates the fixed effects of predictors, random effects across individuals, and flexible curve estimation without distributional constraints. The optimal model obtained a cubic spline with three knot points, yielding a minimum Generalized Cross Validation (GCV) value of 0.980 and evaluated with the Root Mean Square Error (RMSE) value of 0.612. The individual and time random effects are found to be statistically significant (p < 0.05). The findings indicate that the proposed mixed-effects spline offers flexibility in capturing complex trajectories without parametric assumptions. Some highlights proposed in this method are:

• The proposed method integrates truncated spline multivariable estimator within the mixed-effects regression function.
• The parameter estimation uses maximum likelihood method.
• The data uses continuous responses in longitudinal structure.

## Graphical abstract

The graphical abstract was submitted as a separate file.

**Figure:**
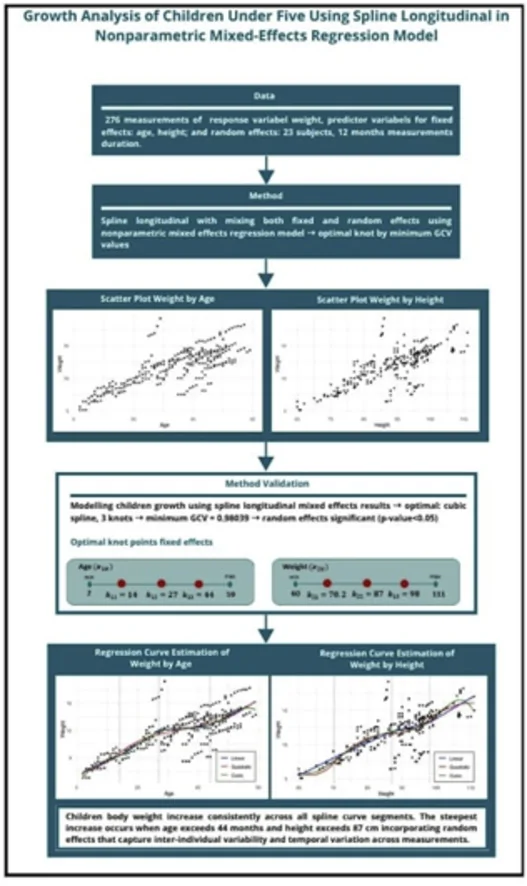

**Specifications table**

**Table:** 

Subject area	Mathematics and Statistics
**More specific subject area**	Statistics modeling
**Name of your method**	Mixed-Effects Longitudinal Spline Regression Model
**Name and reference of original method**	P.J. Diggle, P. Heagerty, K.Y. Liang, S.L. Zeger, Analysis of Longitudinal Data, 2nd ed., Oxford University Press, Oxford, 2002.
Resource availability	This study uses Software R-4.5.2 version to analyze the data by developing a R-code (syntax). The code is available on https://github.com/leylaaisra/spline-mix-effect.

## Background

Regression analysis is a statistical method used to explain the relationship patterns between response and predictor variables [1]. In practice, selecting the appropriate regression approach must be tailored to the characteristics of the data pattern. Nonparametric regression is one of the regression approaches by estimating the regression function [2]. In nonparametric approach, the regression function is estimated without any prespecified parametric form. This method allows the data to determine the shape of the regression curve [3]. Among various nonparametric estimators, the truncated spline estimator stands out in parsimonious modeling of fluctuating data using knot points [4,5]. The piecewise polynomial structure of spline provides both flexibility and interpretability on structural changes across the variables. The truncated spline estimator has been applied in several studies, including analysis on bi-response data [6], time-series data [7], and categorical response data [8].

Another data type that is potentially suitable for modeling with the truncated spline estimator is longitudinal data [9]. Unlike cross-sectional data, longitudinal data allows researchers to observe individual development over time. However, the use of longitudinal data introduces heteroscedasticity that may generate bias in conventional parametric approaches. Moreover, several prior longitudinal studies [10,11] did not explicitly account for within-subject correlations. Although heteroscedasticity can be partially addressed using a covariance matrix as a weighted parameter, this approach still requires the linearity assumption, which is frequently violated in longitudinal data analysis.

The mixed-effects model provides a robust framework to accommodate the within-subject correlation through random effects and fixed effects of predictors within one model [12]. Modeling random effects based on subject identity allows within-subject correlations to be explicitly captured. Mixed-effects models have been examined under both parametric [[13], [14], [15]] and nonparametric frameworks [16,17]. In the nonparametric context, Rice & Wu [16] employed B-Spline functions combined with the expectation-maximization (EM) algorithm and best linear unbiased prediction (BLUP). Heckman et al. [17] applied penalized smoothing using maximum likelihood estimation (MLE) and restricted maximum likelihood estimation (REML). However, both studies primarily utilized nonparametric functions for estimating covariance structures or eigenvalues, rather than directly modeling the regression function nonparametrically. In practice, irregularities in longitudinal data occur not only in time intervals but also in the complex relationship between predictors and responses. Therefore, the present study addresses this gap by directly incorporating a nonparametric estimator into the mixed-effects regression function.

In this study, we develop a spline estimator for mixed-effects longitudinal regression modeling of children’s growth. According to the World Health Organization (WHO), growth standards rely on the complex interaction of anthropometric components, specifically weight, height, and age [18]. Several prior studies about children’s growth [[19], [20], [21]] use cross-sectional studies to analyze the data, which is limited in capturing individual variation and developmental change over time. In contrast, the present study employs a longitudinal approach, enabling repeated observations on the same children across multiple times. Parameter estimation is conducted to derive a model applicable to empirical data. This model captures growth patterns while accounting for random effect variations in one model, which can explicitly describe individual variation across time.

## Method details

### Longitudinal data

Longitudinal data is defined as repeated observations of multiple subjects over a specific duration. This type of data integrates the properties of both cross-sectional and time series data. Cross-sectional data captures a single snapshot across various units. Time series focus on temporal trends for a single entity. Meanwhile, longitudinal data does both various units and temporal trends simultaneously [22]. The general structure of such data is illustrated in Table 1.

**Table 1:** Longitudinal data structure.

Subject (i)	Time (t)	Response Variable ($y_{\mathrm{it}})$	Predictor Variable $\left(x_{1\mathrm{it}}\right)$	…	Predictor Variable $\left(x_{\mathrm{jit}}\right)$
Subject 1	1	$y_{11}$	$x_{111}$	…	$x_{s11}$
	2	$y_{12}$	$x_{112}$	…	$x_{s12}$
	⋮	⋮	⋮	…	⋮
	$m_{1}$	$y_{1m_{1}}$	$x_{11m_{1}}$	…	$x_{s1m_{1}}$
Subject 2	1	$y_{21}$	$x_{121}$	…	$x_{s21}$
	2	$y_{22}$	$x_{122}$	…	$x_{s22}$
	⋮	⋮	⋮	…	⋮
	$m_{2}$	$y_{2m_{2}}$	$x_{12m_{2}}$	…	$x_{s2m_{2}}$
⋮	⋮	⋮	⋮	⋮	⋮
Subject n	1	$y_{n1}$	$x_{1n1}$	…	$x_{sn1}$
	2	$y_{n2}$	$x_{1n2}$	…	$x_{sn2}$
	⋮	⋮	⋮	…	⋮
	$m_{n}$	$y_{nm_{n}}$	$x_{1nm_{n}}$	…	$x_{snm_{n}}$

Let $y_{it}$ represent the response variable for the subject i at time t. The relationship can be expressed as $\left(y_{it},\;x_{it}\right)$ where $x_{itk}$ denotes as predictor j variable for subject i at time t, i=1,2,…,n,t=1,2,…,m,j=1,2,…,s. The relationship can also be formulated as Eq. (1).

(1) $$\begin{array}{c} y_{it}=f\left(x_{jit}\right)+\varepsilon_{it} \end{array}$$

where $f(x_{jit})$ is a function that contains k-th predictor for the corresponding subject and time point, and $\varepsilon_{it}$ is the error of the model that is considered independent.

A fundamental assumption in longitudinal analysis is that observations between different subjects remain independent. The measurements $y_{it}$ within the same individual are correlated. Repeated observations may exhibit mutual correlation driven by between-subject variation. This may happen even if the within-subject errors $\varepsilon_{it}$ are considered independent. The variation accounts for individual differences and rejects the assumption of subject homogeneity. The correlation in longitudinal models is attributed to between-subject variability, while the within-subject error covariance matrix is typically treated as diagonal. It reflects that the within-subject errors are mutually uncorrelated [23].

### Mixed-effects model

A mixed-effects model extends the classical linear framework by simultaneously integrating fixed and random effects into one model [24]. Fixed effects represent parameters that characterize the entire population, whereas random effects account for the inherent variability within that population [25]. In longitudinal analysis, fixed effects capture constant factors across observations, whereas random effects reflect subject-specific variations arising from repeated measurements [26]. The matrix representation of the mixed-effects model is given by Eq. (2) [23]:

(2) $$\begin{array}{c} Y=X\beta +Z\gamma +\varepsilon \; \end{array}$$

where Y is an n×1 response vector for subject i; X is an n×p design matrix for fixed effects predictors; β is a p×1 vector of fixed effect parameters; Z is an n×q design matrix for random effects; γ is a q×1 vector of random effect parameters; and ε is an n×1 vector of residuals. It is assumed that γ and ε are independent, with the assumption $\varepsilon \;∼\;IID\;N(0,\;V_{e}I_{n})$ and $\gamma \;∼\;N\;(0,V_{\gamma }I_{n})$. The mixed-effects model analysis algorithm using maximum likelihood estimation (MLE) by the expectation-maximization (EM) algorithm is formulated with the following steps [27]:

1. Given individual data for subject i conforming to the model in Eq. (2).

2. The complete data log-likelihood function is formulated as in Equation (3):

$\begin{array}{c} \log l\left(\theta \;\right)\;=\sum_{i=1}^{N}\log f\left(y_{i}|\gamma_{i}\right)+\sum_{i=1}^{N}\log f\left(\gamma_{i}\right)\; \end{array}$: (3)

where $N=\sum_{i=1}^{n}n_{i}$ with n denotes the total number of subjects, $n_{i}$ denotes the number of repeated measurements for the subject i, and θ represents the combined parameter vector of the mixed-effects model.

3. Maximize l(θ) by each parameter and equal to zero. Equation (3) is formulated by the EM algorithm by integrating the conditional expectation of the complete-data log-likelihood $Q\left(\theta |\theta^{t}\right)=E_{\gamma |y,\theta^{t}}\left[l(\theta ;y,u)\right].$

4. Updating the variance parameter θ by the result from step 3 to obtain updated parameter estimates $\theta^{t+1}$.

5. Steps 3 and 4 are iterated until convergence is achieved.

### Nonparametric regression model with spline estimator

Regression models are generally categorized into three types based on the specification of the regression curve f(x), which are parametric, nonparametric, and semiparametric. The parametric approach assumes a pre-specified functional form, such as a linear or a polynomial of degree p. The nonparametric regression is employed when the data pattern does not follow any specific parametric form, while semiparametric regression combines both parametric and nonparametric functions. Nonparametric regression is estimated through the regression function, and its model can be expressed as in Eq. (4):

(4) $$\begin{array}{c} y_{i}=f\left(x_{i}\right)+\;\varepsilon_{i};i=1,2,3,\ldots ,n \end{array}$$

where $y_{i}$ is the response variable at the i-th observation, $x_{i}$​ is the predictor variable, $f(x_{i})$ is an unknown regression function, and $\varepsilon_{i}$ is a random error assumed to follow $\varepsilon_{i}∼IID\;N\;\left(0,\sigma^{2}\right)$ [1].

One of the estimators in this framework is spline, a piecewise polynomial characterized by segments joined at specific points called knots. These knots indicate changes in the underlying data patterns [28]. A general nonparametric spline truncated regression of order p is formulated as [23]:

(5) $$\begin{array}{c} y_{i}=\sum_{l=0}^{p}\beta_{l}x_{i}^{l}\;+\sum_{k=1}^{r}\beta_{p+k}{\left(x_{i}-K_{k}\right)}_{+}^{p}+\varepsilon_{i},\;i=1,2,3,\ldots ,n \end{array}$$

with the *truncated* function defined as*:*

$${\left(x_{i}-K_{k}\right)}_{+}^{p}=\left\{ \begin{array}{c} {\left(x_{i}-K_{k}\right)}^{p},\;x_{i}\geq K_{k}\\ 0,\;x_{i}<K_{k}\; \end{array}\right.$$

where $\beta_{j}$ and $\beta_{p+k}$ are spline parameters, $K_{k}$ denotes knot points forming the $K=\{K_{1},\;K_{2},\ldots ,\;K_{r}\}$, and ${\left(x_{i}-K_{k}\right)}_{+}^{p}$ is the truncated polynomial basis function. The nonparametric regression multivariable truncated spline model of order p in matrix form is given by Eq. (6) [29]:

(6) $$\begin{array}{c} y=X\left[K_{1},\;K_{2},\;\ldots ,\;K_{r}\right]\beta +\varepsilon \end{array}$$

Eq. (6) can be written in matrix form as Eq. (7):

(7) $$\begin{array}{c} \left[\begin{array}{c} \begin{array}{c} y_{1}\\ y_{2} \end{array}\\ \vdots \\ y_{n} \end{array}\right]=\left[\begin{array}{cccccccc} 1 & x_{1} & x_{1}^{2} & \ldots & x_{1}^{p} & {\left(x_{1}-K_{1}\right)}_{+}^{p} & \cdots & {\left(x_{1}-K_{r}\right)}_{+}^{p}\\ 1 & x_{2} & x_{2}^{2} & \ldots & x_{2}^{p} & {\left(x_{2}-K_{1}\right)}_{+}^{p} & \cdots & {\left(x_{2}-K_{r}\right)}_{+}^{p}\\ \vdots & \vdots & \vdots & \vdots & \vdots & \vdots & \ddots & \vdots \\ 1 & x_{n} & x_{n}^{2} & \cdots & x_{n}^{p} & {\left(x_{n}-K_{1}\right)}_{+}^{p} & \cdots & {\left(x_{n}-K_{r}\right)}_{+}^{p} \end{array}\right]\left[\begin{array}{c} \beta_{0}\\ \begin{array}{c} \beta_{1}\\ \vdots \\ \beta_{p}\\ \beta_{p+1}\\ \vdots \\ \beta_{p+r\;} \end{array} \end{array}\right]+\left[\begin{array}{c} \varepsilon_{1}\\ \begin{array}{c} \varepsilon_{2}\\ \vdots \\ \varepsilon_{n} \end{array} \end{array}\right] \end{array}$$

where y is an n×1 response vector, $X[K_{1},\;K_{2},\;\ldots ,\;K_{r}]$ is an n×(1+p+r) design matrix, β is a (1+p+r)×1 parameter vector, and εis an n×1 residual vector.

Eq. (5,6) defines nonparametric truncated spline regression for cross-sectional data, where n subjects are observed at one time point. In longitudinal data which n subjects are monitored continuously over a specific duration; the framework must be extended to account for longitudinal structures. Spline truncated estimator in a nonparametric longitudinal multivariable regression model is formulated as follows Eq. (8) [30].

(8) $$\begin{array}{c} y_{it}=\sum_{j=1}^{s}\left(\sum_{l=0}^{p}\beta_{jl}x_{jit}^{l}\;+\sum_{k=1}^{r}\beta_{j\left(p+k\right)}{\left(x_{jit}-K_{jk}\right)}_{+}^{p}\right)+\varepsilon_{it},\;i=1,2,3,\ldots ,n\; \end{array}$$

where j=1,2,…,s is predictor variable and truncated function defined as*:*

$${\left(x_{jit}-K_{ki}\right)}_{+}^{p}=\left\{ \begin{array}{c} {\left(x_{jit}-K_{jk}\right)}^{p},\;x_{jit}\geq K_{jk}\\ 0,\;x_{jii}<K_{jk}\; \end{array}\right.$$

The selection of knot points is crucial for achieving an optimal fit. To maintain model parsimony, Wand [31] suggests utilizing a limited number of knots, while Lee [32] emphasizes that statistical models should prioritize simplicity through optimal knot placement. A standard criterion for identifying these points is based on generalized cross-validation (GCV). The optimal model is based on the minimal value of GCV, formulated as [33]:

(9) $$\begin{array}{c} GCV\;=\frac{n^{-1}{\left(Y-\bar{Y}\right)}^{T}\left(Y-\bar{Y}\right)}{{\left(n^{-1}trace\;\left[I-H\left(K_{jk}\right)\right]\right)}^{2}} \end{array}$$

Here $H\left(K_{jk}\right)=X\left[K_{jk}\right]{\left[(X{\left[K_{jk}\right]}^{T}X\left[K_{jk}\right]\right]}^{-1}X{\left[K_{jk}\right]}^{T}\;$is a hat matrix, and trace$\left[I-H(K_{jk})\right]$ denotes the effective residual degrees of freedom.

Selecting appropriate knot positions is critical for balancing model flexibility and computational efficiency. To guide practical implementation, we outline several knot points selection methods based on sample size and computational resources on Table 2.

**Table 2:** Knot points selection methods.

Number of Datasets	Knot Selection Method	R function	Advantages
Small datasets (N<500)	Trial-and -error	Custom loop over candidate knots	Guarantees near-optimal knot placement
Medium datasets (500≤N≤5000)	Stepwise selection	stepAIC()	Balances fit and parsimony
Large datasets (>5000)	Quantile-based placement	quantile (x,…)	Ensures an equal distribution of data points

Based on Table 2, in this study we choose trial-and -error which offers near-optimal knot placement. This method is suitable for small datasets which can select all the possibilities among datasets. We custom a loop sytax program as a grid-search procedure to account the GCV values for each knot candidate.

### Parameter estimation of mixed-effects longitudinal spline regression

Parameter estimation is conducted via MLE. Based on the longitudinal observations summarized in Table 3, the body weight of children is modeled according to Eq. (2). Assuming the relationship between the response variable and two predictors for the subject i at time t is defined by an unknown function f, the model can be formulated as a nonparametric mixed-effects regression, as shown in Eq. (10):

(10) $$\begin{array}{c} y_{it}=f\left(x_{1it},x_{2it}\right)+Z\gamma +\varepsilon_{it} \end{array}$$

**Table 3:** Descriptive statistics of response and fixed effects predictor variables.

Variable	Symbol	n	Minimum	Maximum	Mean	Standard Deviation
Children’s Weight (kg)	$y_{it}$	276	5.10	19.20	11.71	2.90
Children’s Age (months)	$x_{1it}$	276	2.00	59.00	33.50	14.65
Children’s Height (cm)	$x_{2it}$	276	60.00	111.00	87.74	10.84

If $f(x_{1it},x_{2it})$ is approximated by a multivariable truncated longitudinal spline function, then the nonparametric longitudinal spline regression mixed-effects model is obtained in Eq. (11).

$$y_{it}=\left(f\left(x_{1it}\right)+f\left(x_{2it}\right)\right)+Z\gamma +\varepsilon_{it}$$

(11) $$\begin{array}{c} y_{it}=\left(\sum_{j=1}^{2}f\left(x_{jit}\right)\right)+Z\gamma +\varepsilon_{it} \end{array}$$

where i=1,2,…,23; and t=1,2,…,12.

By substituting Eq. (5) into Eq. (11), the nonparametric longitudinal spline regression mixed-effects multivariable can be expressed in Eq. (12).

(12) $$\begin{array}{c} y_{it}=\sum_{j=1}^{s}\left(\sum_{l=0}^{p}\beta_{jl}x_{jit}^{l}+\sum_{k=1}^{r}\beta_{j\left(p+k\right)}{\left(x_{jit}-K_{jk}\right)}_{+}^{p}\right)+Z\gamma +\varepsilon_{it} \end{array}$$

with the truncated function,

$${\left(x_{jit}-K_{jk}\right)}_{+}^{p}=\left\{ \begin{array}{c} {\left(x_{jit}-K_{jk}\right)}^{p},\;x_{ji}\geq K_{jk}\\ 0,\;x_{ji}<K_{jk}\; \end{array}\right.$$

Eq. (12) can also be written in matrix form as:

(13) $$\begin{array}{c} y_{i}=X_{i}\left[K_{jk}\right]\beta +Z\gamma +\varepsilon_{i} \end{array}$$

Next, the estimated parameter $\hat{\beta }$ is obtained using the MLE method. Calculations are performed to find the expected value and variance of $y_{i}$. It is known that the mixed-effects regression model is normally distributed under the assumptions $\varepsilon \;∼\;IID\;N(0,\;V_{e}I_{n})$ and $\gamma \;∼\;N\;(0,V_{\gamma }I_{n})$. Moreover error ε and random effects γ are independent. Thus, the expected value $y_{i}$ is obtained in Eq. (14–16).

(14) $$\begin{array}{c} E\left(y_{i}\right)\;=E\left(X_{i}\left[K_{jk}\right]\beta +Z\gamma +\varepsilon_{i}\;\right) \end{array}$$

(15) $$\begin{array}{c} E\left(y_{i}\right)=E\left(X_{i}\left[K_{jk}\right]\beta \right)+ZE\left(\gamma \right)+E\left(\varepsilon_{i}\right) \end{array}$$

(16) $$\begin{array}{c} E\left(y_{i}\right)=X_{i}\left[K_{jk}\right]\beta \; \end{array}$$

Furthermore, the variance of $y_{i}$ is obtained by integrating variability between subjects (random effects) and variability within subjects (error), namely:

(17) $$\begin{array}{c} Var\left(y_{i}\right)=\left(Z^{T}V_{\gamma }Z+\;V_{e}\right)I_{n} \end{array}$$

Thus, the distribution of the nonparametric mixed-effects model with a truncated longitudinal spline estimator, where ***y_i_*** is normally marginally distributed with $E\left(y_{i}\right)=X_{i}\left[K_{jk}\right]\beta$ and $Var\left(y_{i}\right)=\left(Z^{T}V_{\gamma }Z+\;V_{e}\right)I_{n}$ can be written as $y_{i}\;∼N(X_{i}\left[K_{jk}\right]\beta ,\left(Z^{T}V_{\gamma }Z+\;V_{e}\right)I_{n}$).

A likelihood function is required that utilizes the probability density function of the normal distribution in the mixed-effects model with a truncated longitudinal spline estimator. The resulting nonparametric mixed-effects model distribution is used to construct the total likelihood function from the marginal likelihood function of the multivariate normal distribution. This marginal likelihood integrates over the random effects. Assuming from Eq. (17), $V^{*}=Z^{T}V_{\gamma }Z+\;V_{e}$, the probability density functions for N-individuals and n-observations are given in Eq. (18).

(18) $$\begin{array}{c} f\left(y_{i},\beta ,\;V^{*}\right)={\left(2\pi \right)}^{-\frac{n}{2}}\;\det{\left(\;V^{*}\right)}^{-\frac{1}{2}}\exp\left(\left(-\frac{1}{2}\right){\left(y_{i}-\;X_{i}\left[K_{jk}\right]\beta \right)}^{T}{\left(V^{*}\right)}^{-1}\left(y_{i}-\;X_{i}\left[K_{jk}\right]\beta \right)\right) \end{array}$$

The marginal likelihood function in Eq. (18) is derived by integrating out all possible values of the random effects γ, a process achieved by taking the product of the joint density and the random effects distribution. Through algebraic reformulation, this marginal density is expressed as the likelihood function shown in Eq. (19).

(19) $$\begin{array}{c} l\left(\beta ,V^{*}|y_{i}\;\right)=\prod_{i=1}^{N}\left(\;{\left(2\pi \right)}^{-\frac{n}{2}}\;\det{\left(\;V^{*}\right)}^{-\frac{1}{2}}\exp\left(\left(-\frac{1}{2}\right)\left(\begin{array}{c} y_{i}^{T}{\left(V^{*}\right)}^{-1}y_{i}-2\beta^{T}X_{i}^{T}\left[K_{jk}\right]\;{\left(V^{*}\right)}^{-1}y_{i}+\\ \beta^{T}X_{i}^{T}\left[K_{jk}\right]{\left(V^{*}\right)}^{-1}X_{i}\left[K_{jk}\right]\beta \end{array}\right)\right)\right)\; \end{array}$$

The natural logarithm of the likelihood function from Eq. (19) can be written as shown in Eq. (20).

(20) $$\begin{array}{c} \ln\left(l\left(\beta ,V^{*}|y_{i}\right)\right)=\ln\left\{\prod_{i=1}^{N}\;\left(\;{\left(2\pi \right)}^{-\frac{n}{2}}\;\det{\left(\;V^{*}\right)}^{-\frac{1}{2}}\exp\left(\left(-\frac{1}{2}\right)\left(\begin{array}{c} y_{i}^{T}{\left(V^{*}\right)}^{-1}y_{i}-2\beta^{T}X_{i}^{T}\left[K_{jk}\right]\;{\left(V^{*}\right)}^{-1}y_{i}\\ +\beta^{T}X_{i}^{T}\left[K_{jk}\right]{\left(V^{*}\right)}^{-1}X_{i}\left[K_{jk}\right]\beta \end{array}\right)\right)\right)\right\} \end{array}$$

To obtain the parameter estimates by maximizing the likelihood function, the partial derivatives of the log-likelihood function in Eq. (20) are taken with respect to each parameter and set to zero. Consequently, the fixed effects parameter estimates are obtained in Eq. (21) as follows:

(21) $$\begin{array}{c} \hat{\beta }=\sum_{i=1}^{N}{\left(X_{i}^{T}\left[K_{jk}\right]{\left(V^{*}\right)}^{-1}X_{i}\left[K_{jk}\right]\beta \right)}^{-1}\sum_{i=1}^{N}\left(X_{i}^{T}\left[K_{jk}\right]\;{\left(V^{*}\right)}^{-1}y_{i}\right)\; \end{array}$$

In Eq. (21), represents the parameter estimation for the fixed effects of child growth using a longitudinal spline within a nonparametric mixed-effects regression model. Furthermore, the parameter estimation for the random effects $V_{\gamma }$ and error $V_{e}$ does not have a closed-form solution because ${\left(V^{*}\right)}^{-1}$ still depends on variance components $V_{\gamma }$**.** Therefore, an iterative solution for the model is required using the EM approach, with the following steps:

1. Construct the posterior covariance matrix:

(22) $$\begin{array}{c} \Omega_{i}^{t}={\left(\frac{Z^{T}Z}{V_{e}}+\frac{I_{n}}{V_{\gamma }}\right)}^{-1} \end{array}$$

and the predicted values of random-effects:

(23) $$\begin{array}{c} \hat{\gamma }=\Omega_{i}^{t}∙\left(\frac{Z^{T}\left(y_{i}-X_{i}\left[K_{jk}\right]\beta \right)}{V_{e}}\right)\; \end{array}$$

2. Calculate the second order expected values for the M-step:

(24) $$\begin{array}{c} E\left[\gamma^{T}\gamma |y_{i}\right]={\hat{\gamma }}^{T}\hat{\gamma }+\Omega_{i}^{t} \end{array}$$

3. Update the parameters $\beta^{t+1}$ and ${V^{*}}^{t+1}$.

4. Calculate the complete-data log-likelihood function, formulated as in Equation (3).

5. Steps 3 and 4 are iterated until convergence is achieved by comparing the log-likelihood before and after the iteration $|\ln L^{t+1}-\ln L^{t}|<10^{-5}$.

### Model evaluation

The selected model is evaluated using GCV value on Eq. (8), *Akaike Information Criterion* (AIC) value, and *Root Mean Square Error* (RMSE) value. The AIC value is calculated based on the likelihood function value and the number of parameters in the model, namely:

(25) $$\begin{array}{c} AIC=-2\ln\left(L\right)+2k\; \end{array}$$

In addition, the evaluation based on the RMSE value can be calculated using the following equation:

(26) $$\begin{array}{c} RMSE=\sqrt{\frac{1}{n}\sum_{i=1}^{n}{\left(y_{i}-{\hat{y}}_{i}\right)}^{2}\;} \end{array}$$

The criteria estimation for each model evaluation are based on the minimum value derived from the comparison of the models used.

## Method validation

### Simulation study

**Fig. 1:**
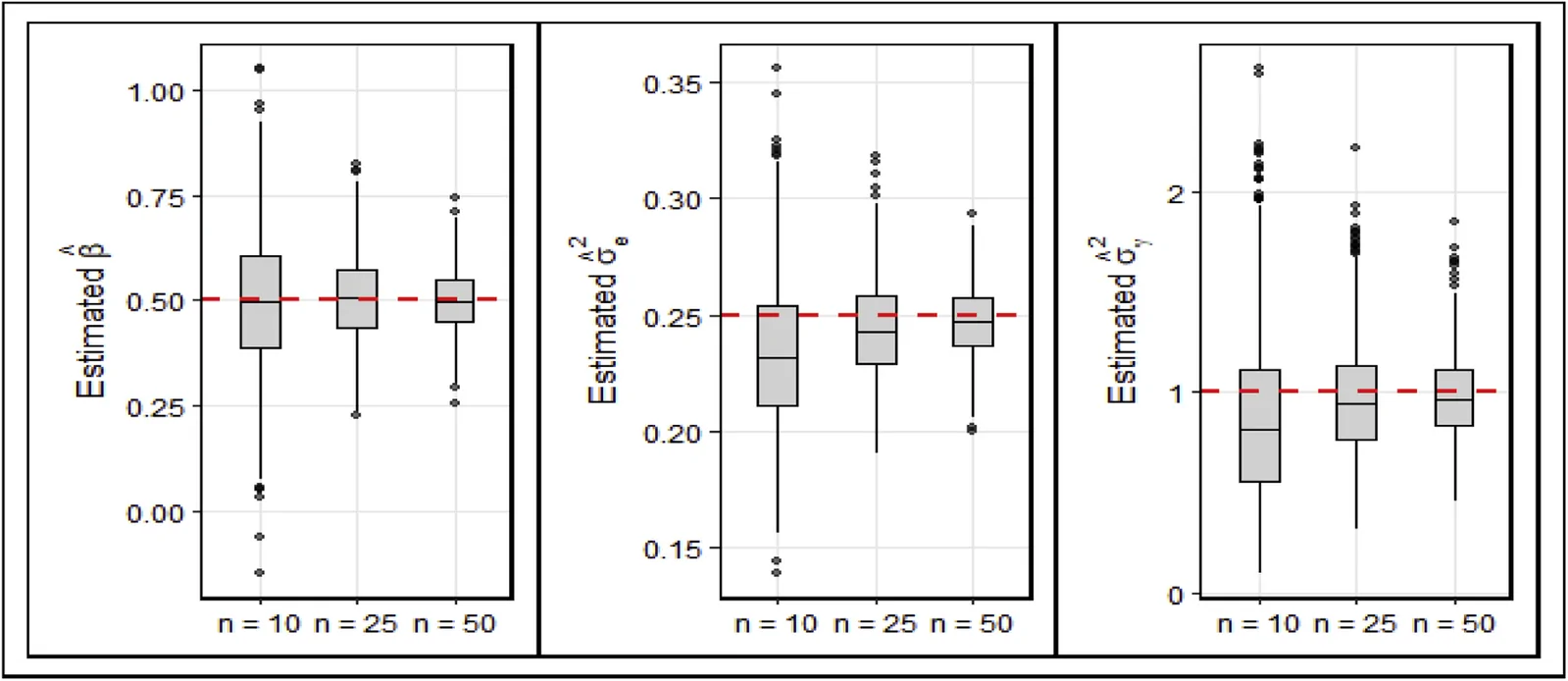
Parameter simulation for n sample.

This study conducted a small-scale simulation study considering sample sizes n∈{10,25,50} across three noise conditions σ∈{0.1,0.5,1.0}. Each subject was measured for 12 times the measurement. The simulation study was performed to investigate parameter recovery and robustness to noise. The true model parameters were set to values similar to those in the real-data illustration. The simulation for three sets of sample size is shown in Fig. 1.

**Fig. 2:**
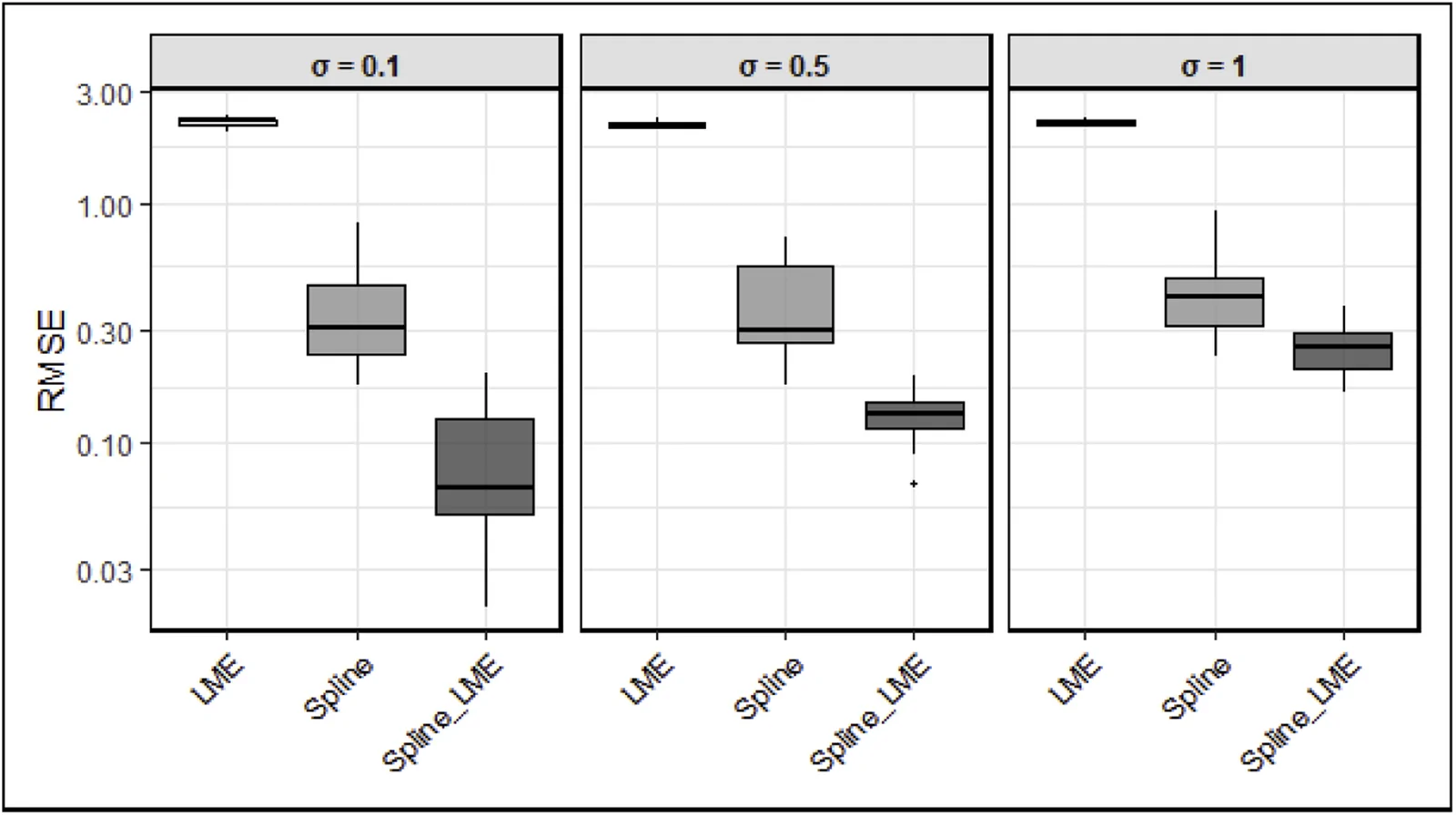
RMSE for simulation noise variations.

Fig. 1 shows that for the fixed-effects coefficient $\left(\hat{\beta }\right)$, the median line across all boxplots align precisely with the reference line (0.5). As the subject count expands, the interquartile range (IQR) and whisker lengths contract continously. The result confirms a substantial gain in estimation precision and steady reduction in sampling variability. A similar trend is observed in the residual variance $\left({\hat{\sigma }}_{e}^{2}\right)$, despite a slight downward shift at n=10, the median converge directly onto true refence line (0.25) at n=50. Meanwhile, the subject-level random-effects variance $\left({\hat{\sigma }}_{\gamma }^{2}\right)$ shows a mild downward bias under small sample contions at n=10. The medians shift upward to match the true parameter as the sample size increases. The simulation confirms the proposed estimation procedure recovers all underlying data-generating without structural bias. Moreover, simulations were also conducted under various noise conditions, as presented in Fig. 2.

Fig. 2 shows that an increase in noise variance naturally elevates the RMSE values across all models. However, the proposed method proves to be the most robust, consistently producing the lowest error and maintaining superior stability at every noise level. This confirms that integrating the spline approach with linear mixed-effects (LME) modeling provides strong protection against data noise variability.

### Data description

The data used in this study were derived from data collected at Bontoala Integrated Healthcare Center in Makassar City in 2024. The dataset consists of longitudinal measurements of 23 children aged 0–60 months, monitored over a 12-month observation period, resulting in a total of 276 data points. As detailed in Table 3, the research variables include children’s weight $\left(y_{it}\right)$ as the response variable, age $\left(x_{1it}\right)$ and height $\left(x_{2it}\right)$ as fixed effect predictors. This study used R software to analyze the data. The mixed-effects spline regression method was implemented using the nlme 3.1.168 version for fitting the nonparametric mixed-effects model and ggplot2 4.0.1 version to visualize the estimated curves.

Table 3 presents the descriptive statistics of the variables used in this study. The results show that the standard deviation values are in the high range. The variability observed across all the three variables based on standard deviation indicates a strong potential for between-subject heterogeneity. The wide range of weight (5.10–19.20 kg), age groups (2–59 months), and height (60.0–111.0 cm) provides a sufficiently heterogenous dataset to evaluate the model’s ability to capture individual-level variation over time. These results may identify the growth trajectories of children over a specific duration.

**Fig. 3:**
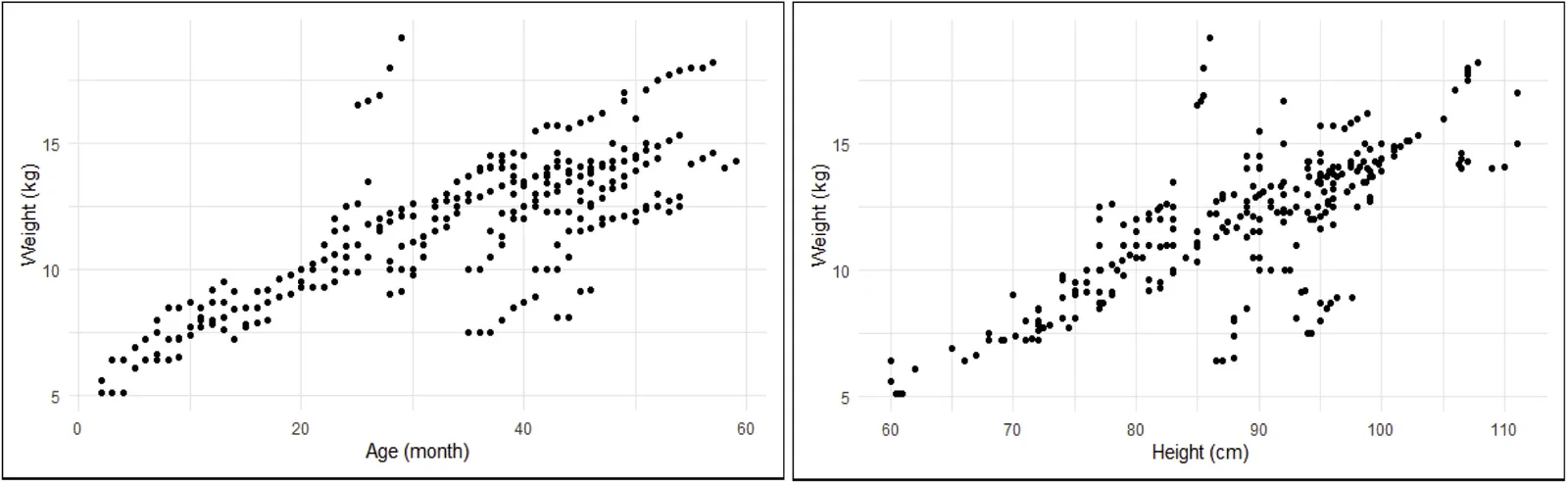
Plot of changes in children’s weight ($y_{it}$) against: (a) children’s age ($x_{1it}$) and (b) children’s height ($x_{2it}$).

Data visualizations in Fig. 3(a) and Fig. 3(b) reveal an increasing trend in the relationship between children’s weight and each predictor variable across individual subjects. However, the relationship patterns are not entirely linear, as several data points deviate from the straight-line trend. Additionally, both Fig. 3(a) and Fig. 3(b) exhibit sharp fluctuations at certain points. Conventional parametric regression lines fail to capture data points situated far from the majority of the data distribution. This condition indicates that a nonparametric approach is appropriate for modeling the data.

### Nonparametric spline longitudinal mixed-effects modeling for growth data

The child’s growth is modeled using a nonparametric longitudinal spline regression with mixed-effects at each of the linear, quadratic, and cubic orders. Optimal knot point selection was performed using the GCV criterion on a model with a random intercept as an initial exploratory stage. The optimal knot points were subsequently incorporated into the final model by simultaneously integrating individual random intercepts and time random slopes.

**Fig. 4:**
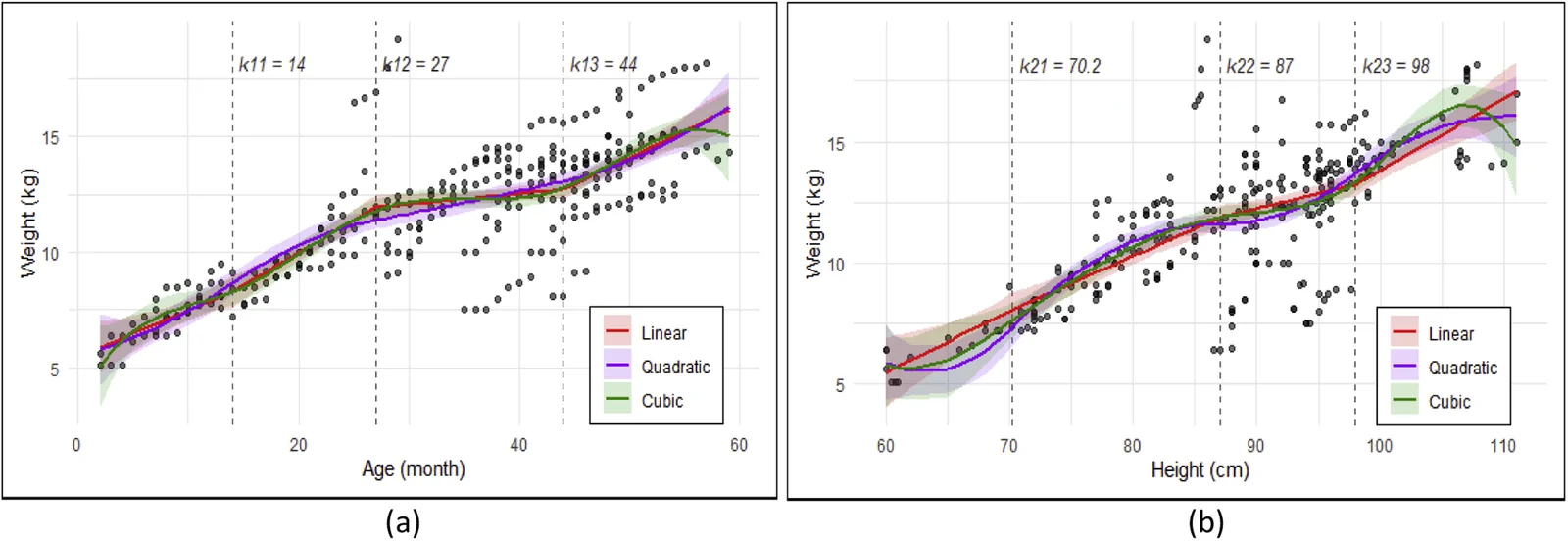
Estimated growth curves with confidence bands in every order between response and each predictor variable: (a) children’s weight $\left(y_{it}\right)$ and children’s age $\left(x_{1it}\right)$, (b) children’s weight $\left(y_{it}\right)$ and children’s weight $\left(x_{2it}\right)$.

**Fig. 5:**
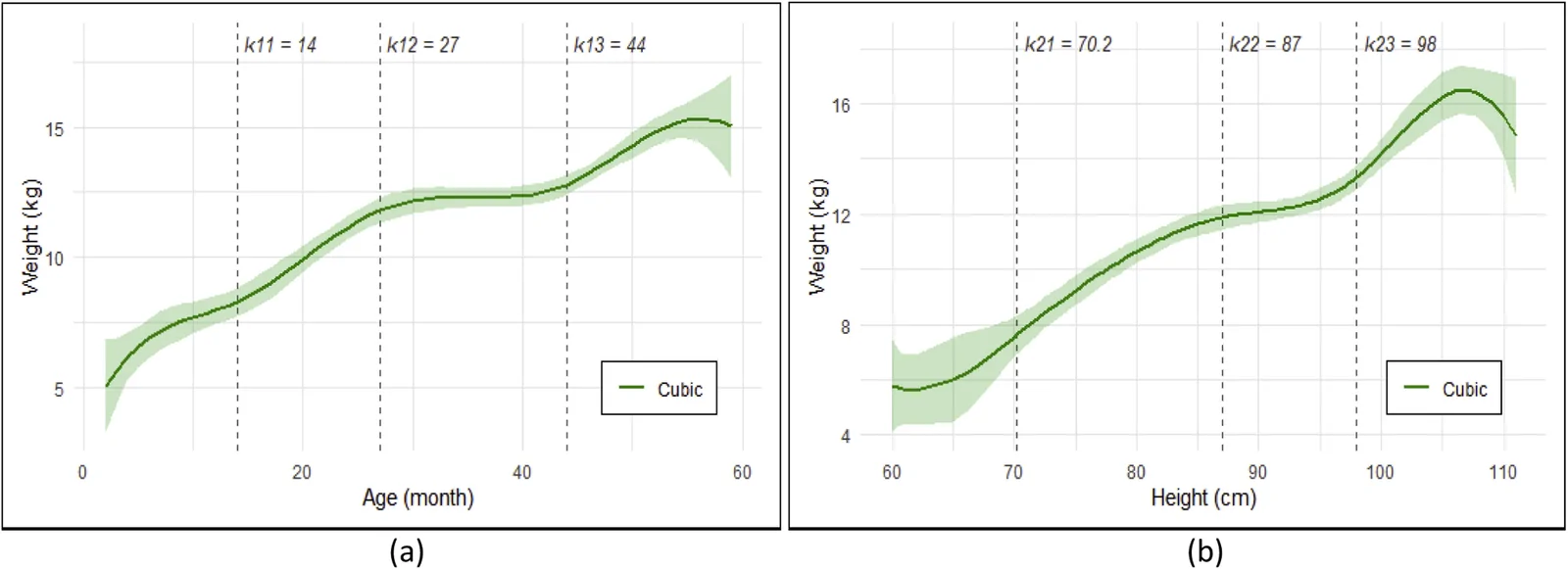
The optimal estimated curves with confidence bands of changes in growth data between response and each predictor: (a) children’s weight $\left(y_{it}\right)$ and children’s age $\left(x_{1it}\right)$, (b) children’s weight $\left(y_{it}\right)$ and children’s weight $\left(x_{2it}\right)$.

The analysis results presented in Table 4 indicate that the model with the minimum GCV value is simultaneously located at the cubic order model with 3 knot points. The knot points for the variable $x_{jit}$ with the number of predictor variables j= 1,2 and the number of knot points tested k= 1,…,4. From Table 4, the minimum GCV value of 0.980 is located at the knot points for the variable $x_{1it}$**,** namely $k_{11}=$ 14, $k_{12}=$ 27, and $k_{13}=$ 44 and knot points for the variable $x_{2it}$**,** namely $k_{21}=$ 70.2, $k_{22}=$ 87, and $k_{23}=$ 98. The estimated curves with three knot points for each specific order are presented in Fig. 4, while Fig. 5 displays the optimal regression curve.

**Table 4:** Comparison of multivariable spline longitudinal GCV and AIC values.

Order	Number of Knots	Knot Point $x_{1it}$				Knot Point $x_{2it}$				GCV	AIC
		$k_{11}$	$k_{12}$	$k_{13}$	$k_{14}$	$k_{21}$	$k_{22}$	$k_{23}$	$k_{24}$		
1	1	26				77.3				1.0619	920.6669
	2	18	36			65	95.3			1.0014	914.0438
	3	14	27	44		70.2	87	98		0.9880	909.7020
	4	12	24	36	48	67	74	97	107	1.0545	924.1777
2	1	26				77.3				0.9936	911.4752
	2	18	36			65	95.3			1.0077	915.2052
	3	14	27	44		70.2	87	98		1.0267	918.1587
	4	12	24	36	48	67	74	97	107	1.0166	915.8741
3	1	26				77.3				1.0069	915.3868
	2	18	36			65	95.3			1.0150	920.2688
	3	14	27	44		70.2	87	98		0.9804**	908.4874**
	4	12	24	36	48	67	74	97	107	1.0225	921.2890

Based on Fig. 5, both predictors show a general upward trend, with the spline model capturing different behaviors across each segment. As shown in Fig. 5(a), the response variable for weight $\left(y_{\mathrm{it}}\right)$ increases significantly within the age $\left(x_{1\mathrm{it}}\right)$ segments bounded by the first two knots, $k_{11}$ and $k_{12}$. After these knots, the trajectory moves at a constant rate toward the third knot $(k_{13}).$ The cubic curve begins to bend downward compared to the linear and quadratic curves on Fig. 5(a) after reaching the third knot.

A similar segmented pattern is observed for the height variable $\left(x_{2it}\right)$ in Fig. 5(b). The weight increases steadily within the segments defined by knots $k_{21}$ and $k_{22}$. However, the growth rate when approaching the third knot $k_{23}$ is faster than that observed in age-based curve. Furthermore, the cubic curve reverses direction and declines sharply after reaching this third knot. Based on Fig. 4, Fig. 5, the confidence bands widen at the terminal ends and narrow near the knot points. This indicates that population trajectory estimation is subject to higher uncertainty at the study boundaries, while exhibiting high precision and confidence near the knots where non-linear growth changes occur.

The optimal knot points with the minimum GCV values selected in Table 4 were then used for simultaneous data modeling by incorporating fixed and random effects. Based on Eq. (12), the mixed-effects longitudinal spline multivariable regression model for cubic order with three knot points is obtained as Eq. (27):

(27) $$\begin{array}{c} y_{it}=\sum_{j=1}^{s}\left(\sum_{l=0}^{3}\beta_{jl}x_{jit}^{l}+\sum_{k=1}^{3}\beta_{j\left(p+k\right)}{\left(x_{jit}-K_{jk}\right)}_{+}^{p}\right)+Z\gamma +\varepsilon_{it} \end{array}$$

The statistical testing on Table 5 indicates that the predictor variable $x_{1it}$ has no statistically significant at α=0.05 on the response variable across all basis function $\left({\hat{\beta }}_{j(p+k)}>0.05\right)$. Meanwhile, for predictor variable $x_{2it}$, two spline basis function parameters show statistically significant, which are $\left({\hat{\beta }}_{25}<0.05\right)$ and $\left({\hat{\beta }}_{26}<0.05\right)$.The 95% confidence interval for these parameters are [0.0002, 0.0029] and [−0.0054, −0.0006], respectively, neither of which spans zero. These results confirm the presence of significant localized non-linear trends and slope changes in $x_{2it}$ beyond its corresponding knot locations and the knot point. Furthermore, the estimation of random effects parameters is shown on Table 6.

**Table 5:** Estimation results of fixed effect parameters.

Variable	Parameter	Estimated Coefficient	SE	CI-Lower	CI-Upper	p-value
(Intercept)	${\hat{\beta }}_{0}$	−446.7134	568.3076	−1566.2000	672.7708	0.4326
$x_{1it}$	${\hat{\beta }}_{11}$	0.2658	0.5616	−0.8404	1.3720	0.6364
	${\hat{\beta }}_{12}$	−0.0029	0.0493	−0.1001	0.0942	0.9526
	${\hat{\beta }}_{13}$	0.0001	0.0014	−0.0026	0.0028	0.9438
	${\hat{\beta }}_{14}$	−0.0002	0.0017	−0.0037	0.0032	0.8867
	${\hat{\beta }}_{15}$	0.0001	0.0007	−0.0012	0.0015	0.8231
	${\hat{\beta }}_{16}$	−0.0003	0.0008	−0.0018	0.0012	0.6941
$x_{2it}$	${\hat{\beta }}_{21}$	20.1884	25.1057	−29.2663	69.6431	0.4221
	${\hat{\beta }}_{22}$	−0.2996	0.3678	−1.0241	0.4250	0.4162
	${\hat{\beta }}_{23}$	0.0015	0.0018	−0.0021	0.0050	0.4102
	${\hat{\beta }}_{24}$	−0.0019	0.0021	−0.0059	0.0021	0.3534
	${\hat{\beta }}_{25}$	0.0016	0.0007	0.0002	0.0029	0.0218
	${\hat{\beta }}_{26}$	−0.0030	0.0012	−0.0054	−0.0006	0.0138

**Table 6:** Estimation results of random effect parameters.

Random Effects	Parameter	${\hat{\sigma }}_{\gamma }^{2}$	LRT $(G^{2})\;$	p-value
Subject (i)	${\hat{\gamma }}_{i}$	6.1915	160.5726	<0.05**
Time $\left(t_{i}\right)$	${\hat{\gamma }}_{t}$	0.0918	157.2699	<0.05**

From the parameter estimation results in Table 5, Table 6, the mixed-effects longitudinal cubic spline regression model estimation is obtained, namely:

(28) $$\begin{array}{c} {\hat{y}}_{it}=-446.7134\;+\;0.2658x_{1it}-\;0.0029x_{1it}^{2}+\;0.0001x_{1it}^{3}-\;0.0002{\left(x_{1it}-\;14\right)}_{+}^{3}\\ +\;0.0001{\left(x_{1it}-\;27\right)}_{+}^{3}-\;0.0003{\left(x_{1it}-\;44\right)}_{+}^{3}+\;20.1884x_{2it}-\;0.2996x_{2it}^{2}+\;0.0015x_{2it}^{3}\\ -\;0.0019{\left(x_{2it}-70.2\right)}_{+}^{3}+\;0.0016{\left(x_{2it}-87.0\right)}_{+}^{3}-\;0.0030{\left(x_{1it}-\;98.0\right)}_{+}^{3}+{\hat{\gamma }}_{i}+{\hat{\gamma }}_{t}\;\# \end{array}$$

Eq. (28) presents the mixed-effects longitudinal cubic spline regression model, which divides the predictor range into four segments based on three optimal knot points. For the age variable $\left(x_{1it}\right)$, beyond the first knot $\left(k_{11}\right)$, the negative truncated coefficient indicates a slight deceleration in weight gain between 14–27 months. Between the second $\left(k_{12}\right)$ and third knot $\left(k_{13}\right)$, the cumulative cubic effect approaches zero, reflecting a nearly constant growth rate between 27–44 months. These conditions are consistent with Fig. 4(a). A further deceleration compared to earlier segments happens after the third knot.

**Fig. 6:**
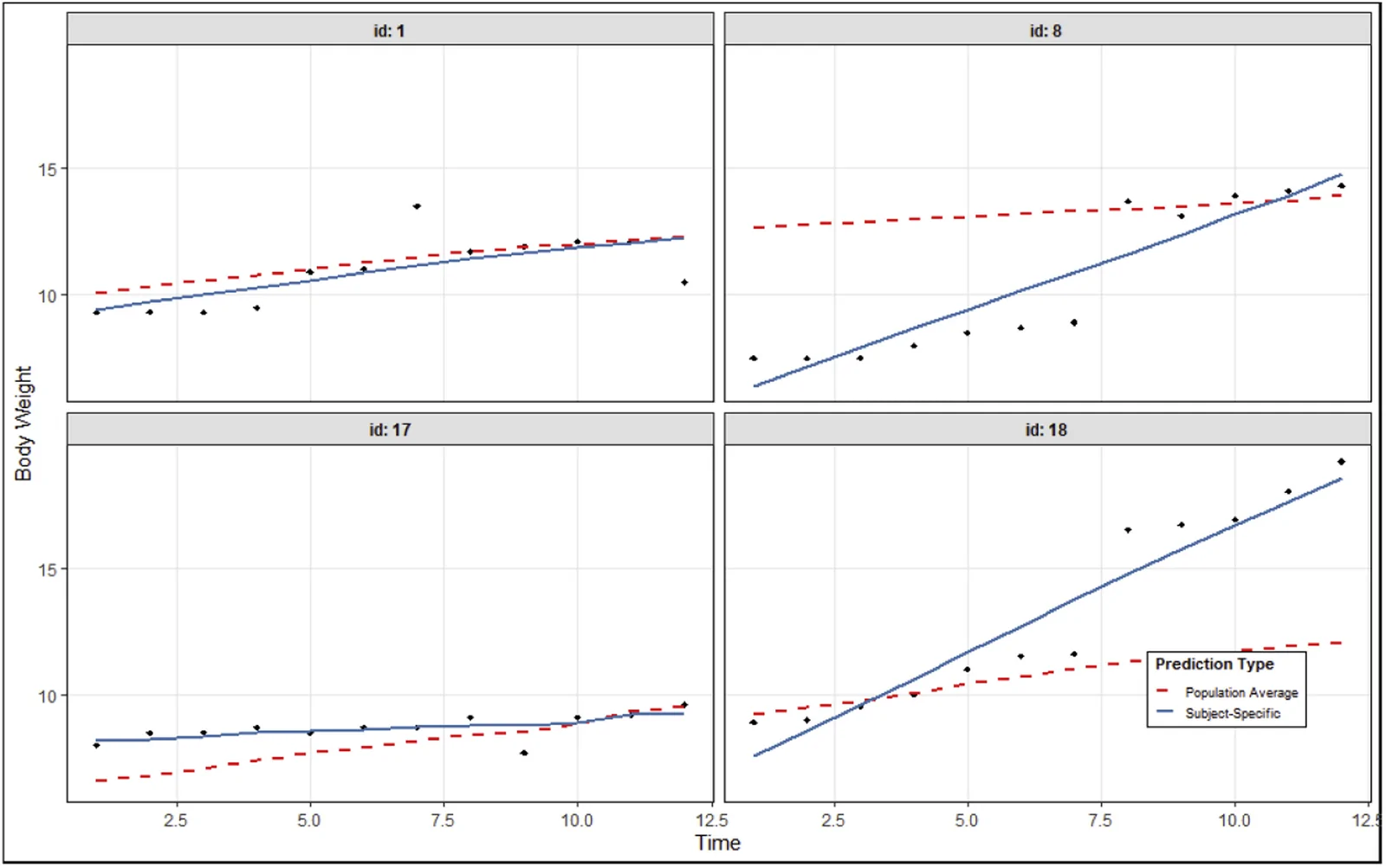
Subject-specific trajectories compare to population curve.

For the height variable $\left(x_{2it}\right)$, beyond the first knot $\left(k_{21}\right)$ show the negative truncated coefficient, which represents a deceleration in weight gain for heights around 70.2–87 cm. This is followed by an accelerated growth rate within the 87–98 cm range $\left(k_{22}\right)$. Furthermore, past the third knot $\left(k_{23}\right)$ shows a negative cumulative cubic effect, which indicates a deceleration in weight gain for children’s height exceeding 98 cm. These conditions are depicted in Fig. 4(b). These result are obtained by accommodating the effects of individual variations in random-effects $\hat{\gamma }$. The subject-specific trajectories is depicted on Fig. 6.

Fig. 6 illustrates that Subject 8 (ID: 8) has the lowest baseline value relative to the population mean, while Subject 17 (ID: 17) shows a higher baseline value. Additionally, Subject 18 (ID: 18) exhibits the fastest trajectory rate, whereas Subject 17 displays the slowest growth trajectory.

The mixed-effects model also incorporates individual and temporal random effects, with variance components summarized in Table 6. An individual random variance component ${\hat{\sigma }}_{\gamma_{i}}^{2}=$ 6.19,146 indicates substantial differences in growth trajectories among individuals. Meanwhile, the smaller temporal variance ${\hat{\sigma }}_{\gamma_{t}}^{2}=$ 0.09,184 suggests that monthly within-subject fluctuations remain relatively stable. Despite this difference, the likelihood ratio test (LRT) confirms that both individual and temporal random effects significantly contribute to the model.

**Fig. 7:**
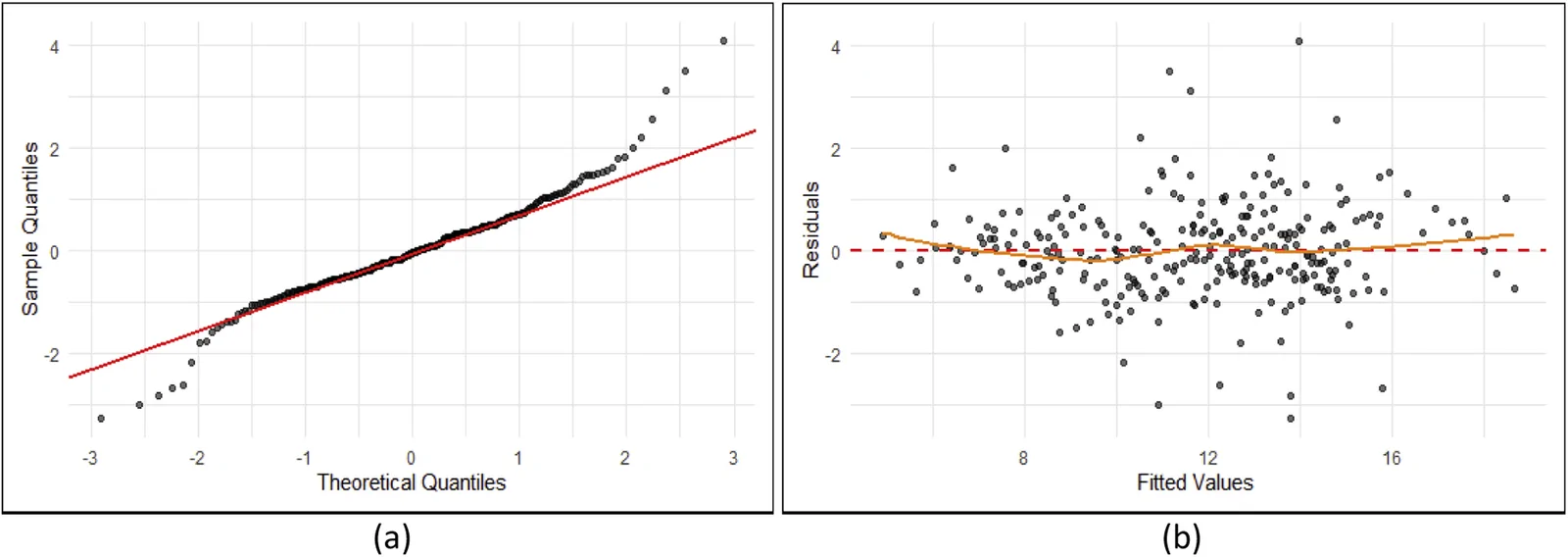
Diagnostic model of: (a) normality residuals, (b) homogenous variance residuals.

To validate the goodness-of-fit and verify that core statistical assumptions hold for the estimated mixed-effects longitudinal spline model, visual residual diagnostics were evaluated (Fig. 7). Model diagnostics were performed by investigating error normality and the assumption of homoscedasticity.

The normal Q-Q Plot on Fig. 7(a) shows that the central bulk of sample quantiles strictly adheres to the theoretical normal line. Although minor deviations are observed at the extreme upper and lower tails, a common characteristic in empirical longitudinal anthropometric data, the overall pattern confirms that the residual distribution sufficiently approximates normality. Collectively, these diagnostic checks verify the statistical validity and robustness of the proposed framework. Furthermore, in the *Residuals* vs. *Fitted Values* plot on Fig. 7(b), the residual points are randomly and uniformly distributed around the zero-reference line (y=0). The lowess smoothing line (orange curve) aligns closely with (y=0), confirming that the assumptions of homoscedasticity are well satisfied.

### Model evaluation

The model evaluation based on the Akaike Information Criterion (AIC) is shown in Table 4. The cubic spline model with three knots yielded the lowest AIC among the orders. This result indicates that the cubic spline model with three knots achieves the best balance between goodness of fit and model simplicity. Furthermore, the selected model was compared against conventional models using in-sample functional fit root mean square error (RMSE). The results obtained are detailed in Table 7.

**Table 7:** Model evaluation.

Model	RMSE
Spline Longitudinal Model	1.7031
Mixed-Effects Model	0.6309
Mixed-Effects Longitudinal Spline Model	0.6122**

Table 7 shows a comparison of RMSE values for cubic orders of the spline longitudinal model, the mixed-effects model, and the mixed-effects longitudinal spline model. The results show that the mixed-effects longitudinal spline model has the smallest RMSE value among the three models. To quantify the uncertainty of the RMSE estimates between the mixed-effects model and the mixed-effects longitudinal spline model, a bootstrap analysis was conducted using subject-level resampling with 5000 replications. The bootstrap preserves the correlation structure inherent in the longitudinal data. The results indicate that the proposed spline model yielded a mean RMSE of 0.6099 (95% CI: 0.4848–0.7344), while the conventional model yielded a mean RMSE of 0.6283 (95% CI: 0.4967–0.7627). The difference in RMSE between the two models had a mean of 0.0184, with a 95% confidence interval of (−0.0077, 0.0459). The results show that the bootstrap of confidence interval includes zero. Rather than offering a significant reduction in prediction error, the primary advantage of the proposed framework is in its structural flexibility to capture non-linear trajectories without strict parametric assumptions.

The use of nonparametric models in mixed effects not only accounts for variation among subjects but also enables the creation of a mathematical model that describes a data does not follow a parametric curve. This indicates that nonparametric modeling is more flexible and performs better by accounting for the random effects of potential variation among subjects over time. Practical measurement errors in anthropometric variables (weight, height, age) also deserve consideration. In longitudinal studies, random errors often stem from uncalibrated instruments, improper subject positioning, or recording mistakes. In this study, we assume a classical measurement error structure, where the observed measurement values differ from the true underlying trajectory by an independent random noise with zero mean. This measurement error is considered within the residual variance component of the mixed-effects model.

Additionally, this study employs an exhaustive grid search (trial-and-error) method to select optimal knot locations, which is computationally time-consuming for large datasets or models with numerous variables. Alternative knot selection strategies can be considered, namely quantile-based knot placement, which distributes knots across specific sample percentiles. Unlike the trial-and-error approach, which evaluates all possible knot combinations across the data range, the quantile-based method relies solely on data density. Another alternative is sequential knot placement, which uses a stepwise procedure by initially placing multiple candidate knots across the range and iteratively removing those with the smallest contribution to model fit.

This method can be expanded by using a penalized spline estimator, where the number of knots is specified and the smoothness of the curve is controlled by a penalty. This method aligns with the Generalized Additive Mixed Model (GAMM), which incorporates a penalty function into the non-linear effects between variables but may more time consuming due to penalty consideration. Beyond continuous outcomes, expanding this mixed-effects longitudinal spline framework to noncontinuous responses presents a promising avenue for future research. Extending the current model to Generalized Linear Mixed Models (GLMMs) with spline estimator would enable the handling of count (e.g., poisson or negative binomial), and categorical data (e.g., logistics). Furthermore, while the present formulation assumes a homogeneous random effects structure across all subjects, real-world growth trajectories often exhibit heterogeneity. Future studies can consider adding nonparametric functions on random effects variance estimation.

### Limitations

1. This method can only be used for response variables and fixed effects predictors for continuous data.
2. he try-error knot points used in the implementation of datasets are k=1,…,4.
3. The estimation method used in the mixed-effects longitudinal spline regression model is Maximum Likelihood Estimation (MLE).

## Related research article

A. Islamiyati, A. Kalondeng, M. Zakir, S. Djibe, U. Sari, Detecting age prone to growth retardation in children through a bi-response nonparametric regression model with a penalized spline estimator, Iran. J. Nurs. Midwifery Res. 29 (5) (2024) 549–554. https://doi.org/10.4103/ijnmr.ijnmr_342_22.

## Ethics statements

This research involved human subjects, and in the data collection process, it has gone through Research Permit No 11,097/UN4.11/PT.01.04/2024 from the Department of Statistics.

## CRediT author statement

**Isra Leyla Bangsawang**: Conceptualization, Data Curation, Formal Analysis, Writing – Original Draft. **Anna Islamiyati**: Methodology, Software. **Erna Tri Herdiani**: Investigation.

## Declaration of competing interest

The authors declare that they have no known competing financial interests or personal relationships that could have appeared to influence the work reported in this paper.

## Data Availability

Data will be made available on request.
